# Dietary L-arabinose-induced gut dysbiosis exacerbates *Salmonella* infection outcome

**DOI:** 10.1128/msystems.00522-24

**Published:** 2024-07-09

**Authors:** Jingchen Yu, Huang Tang, Ning Zhou, Zuoqiang Wang, Wanqiu Huang, Yana Chen, Danni Wang, Jinjing Ni, Jie Lu, Yu-Feng Yao

**Affiliations:** 1 Laboratory of Bacterial Pathogenesis, Shanghai Institute of Immunology, Shanghai Jiao Tong University School of Medicine, Shanghai, China; 2 Department of Pediatrics, Anhui Provincial Hospital, The First Affiliated Hospital of USTC, Hefei, Anhui, China; 3 Department of Infectious Diseases, Ruijin Hospital, Shanghai Jiao Tong University School of Medicine, Shanghai, China; 4 State Key Laboratory of Microbial Metabolism, School of Life Sciences and Biotechnology, Shanghai Jiao Tong University, Shanghai, China; 5 Shanghai Key Laboratory of Emergency Prevention, Diagnosis and Treatment of Respiratory Infectious Diseases, Shanghai, China; University of California San Diego, La Jolla, California, USA

**Keywords:** *Salmonella*, microbiota, L-arabinose, metabolism

## Abstract

The gut microbiota is essential for providing colonization resistance against pathogens. Dietary sugars markedly shift the composition of the intestinal microbiota and alter host susceptibility to enteric infections. Here, we demonstrate the effect of L-arabinose on bacterial infection by using a mouse infection model with *Salmonella enterica* serovar Typhimurium (*S*. Tm). In the presence of microbiota, L-arabinose induces a dramatic expansion of *Enterobacteriaceae*, thereby decreasing the microbiota diversity and causing more severe systemic infection. However, L-arabinose supplementation does not alter the disease progression of *Salmonella* infection in a microbiota-depleted mouse model. More importantly, short-term supplementation of L-arabinose fails to exert anti-diabetic effects in *Salmonella*-infected hyperglycemia mice and still promotes infection. Overall, our work reveals that a high intake of dietary L-arabinose supports a bloom of *Enterobacteriaceae* in *Salmonella*-infected gut, further accelerating the process of systemic infection.

**IMPORTANCE:**

L-arabinose is a promising natural sweetener and food additive for the regulation of hyperglycemia. Since diabetic subjects are more susceptible to infections, the safety of dietary L-arabinose in diabetic patients experiencing infection remains a concern. Our findings reveal that L-arabinose exacerbates *Salmonella* infection outcome by inducing gut microbiota dysbiosis in mice. High dietary intake of L-arabinose may be deleterious for diabetic individuals undergoing infection.

## INTRODUCTION

The scavenging of nutrients is the first step for pathogens to occupy and proliferate in the gut, and microbiota composition, host genetic conditions, or antibiotic treatment influences the course of enteric infections (1). The accessibility of dietary sugars by pathogenic bacteria is crucial for successful infection of the host (2). To achieve a unique niche in the lumen, pathogens must successfully compete with the microbiota for nutrients. For example, two epidemic ribotypes of *Clostridium difficile*, RT027 and RT078, can metabolize low concentrations of dietary trehalose. Challenging mice with trehalose increases disease severity in humanized microbiota mice (3). Similarly, *Citrobacter rodentium* utilizes galacturonic acid as a carbon source to aid the initial pathogen expansion in the mouse gut (4). Specifically, the pathogen may take advantage of nutrients that are not consumed by the normal microbiota (5). Thus, altering the availability of nutrients may create a new nutrient-defined intestinal niche, which promotes or restricts the growth of invading bacteria.

*Salmonella enterica* serovar Typhimurium (*S*. Tm) is a facultative intracellular pathogen that produces both localized gastroenteritis and disseminated systemic disease in humans and animals (6). During infection, *Salmonella* can take up and metabolize a wide array of carbohydrates and gluconeogenic substrates (7). Once in the gut, *S*. Tm uses several short-chain fatty acids, such as acetate, propionate, and butyrate to colonize the lumen (2). In addition, inflammation-derived electron acceptors tetrathionate and nitrate can also be used by *S*. Tm for anaerobic respiration, which supports the utilization of poorly fermentable carbon sources, such as succinate, lactate, and ethanolamine in the host gut (8–10). Together, *S*. Tm takes advantage of its diverse metabolism strategies to outgrow resident microbiota and exploit intestinal nutrient niches.

Simple sugar metabolism plays a crucial role in the ecophysiology of the human gut microbiota, which overrides host genetic effects (11, 12). L-arabinose, an abundant monosaccharide in plants, has been proven to selectively inhibit intestinal sucrase activity in an uncompetitive manner and suppress diet-induced obesity in humans (13). L-arabinose is poorly absorbed in the human gut, however, many gut bacteria use it as a source of carbon and energy (14). For example, L-arabinose suppresses diet-induced obesity in mice in the presence of sucrose and serves as a modulator to preserve host gut-microbiota homeostasis in a healthy gut (15). However, the beneficial effects of L-arabinose have mostly been investigated on uninfected humans or animals (16, 17). The role of L-arabinose in infected or inflamed guts remains largely unknown.

L-arabinose is utilized by both pathogens and commensals to establish or maintain host colonization. It seems that L-arabinose modulates enteric infections, serving as both an environmental signal and a nutrient in the host intestine. For example, L-arabinose uptake in *S*. Tm is mediated by AraE permease (18). The expression of the *araBAD* operon, which encodes enzymes for metabolizing L-arabinose to D-xylulose-5-phosphate, is induced by intracellular L-arabinose (19). Notably, L-arabinose catabolism confers a competitive advantage for *Salmonella* in the gastrointestinal tract and promotes the rapid emergence of superspreaders (20). Commensal species, such as *Escherichia coli* and *Bacteroides thetaiotaomicron* also compete with L-arabinose for carbon utilization, which modifies the within-host environment in inflamed guts and shapes different infection outcomes (5, 21, 22). Since L-arabinose is a dietary supplement for diabetic patients, who are usually associated with enteric infections and microbiota dysbiosis (23), it is urgent to clarify the role of L-arabinose in enteric infections.

In this study, we showed that L-arabinose exacerbated intestinal and systemic inflammation after *Salmonella* infection. Particularly, L-arabinose-treated mice resulted in a loss of gut microbiome diversity and overgrowth of *Enterobacteriaceae* upon *Salmonella* infection. In addition, L-arabinose also reduced the survival rate of *Salmonella-*infected hyperglycemic mice. Taken together, our findings suggest that a high-L-arabinose diet triggers gut microbiota dysfunction and promotes systemic inflammation upon *Salmonella* infection.

## RESULTS

### L-arabinose exacerbates *Salmonella* infection outcome in a mouse typhoid model

**Fig 1:**
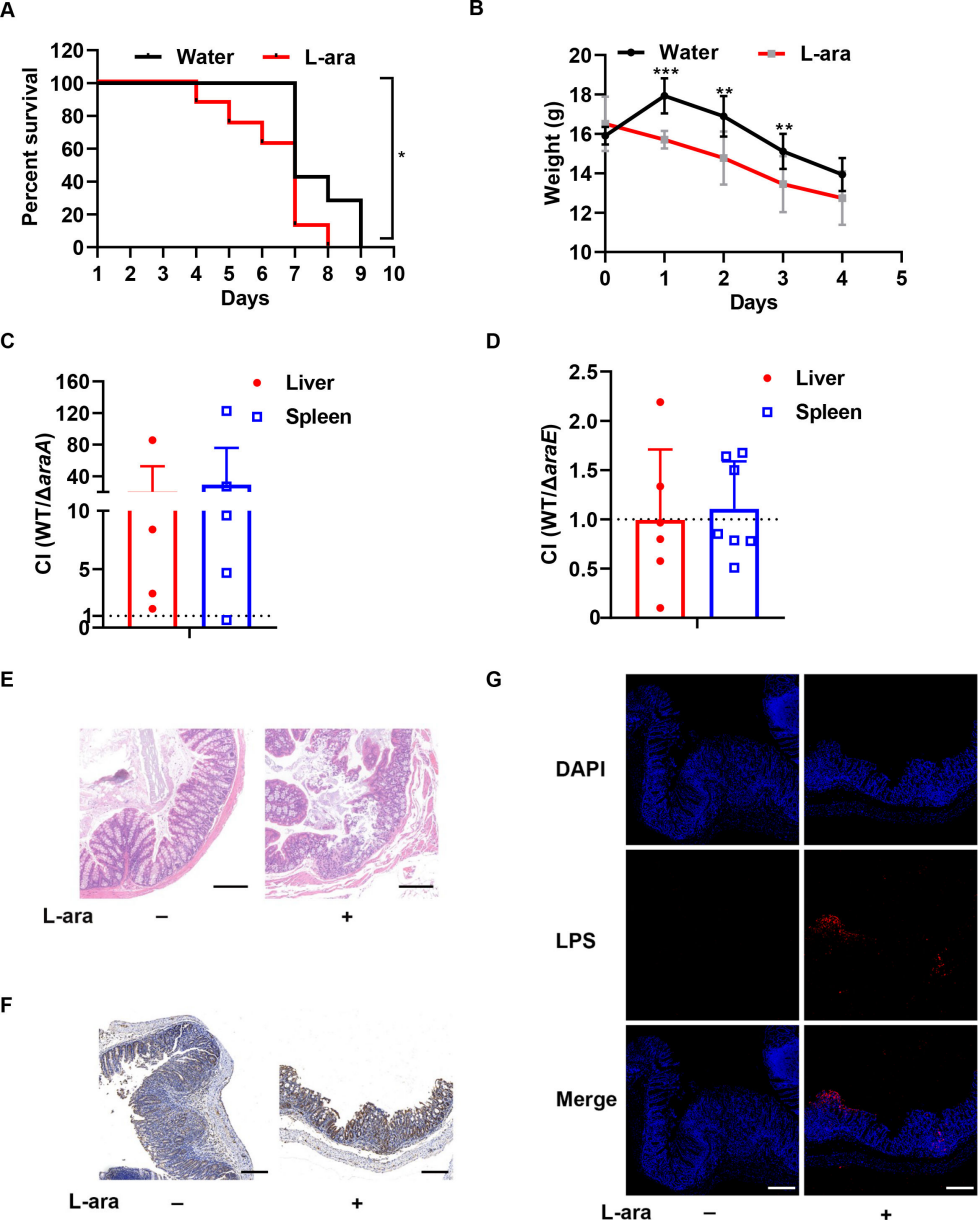
L-arabinose exacerbates *Salmonella* infection in conventional mice. (**A**) Survival curve of conventional mice infected with *S*. Tm and supplemented with water or 4.5% (m/v) L-arabinose (L-ara)-containing water. *, *P* < 0.05, log-rank test. (**B**) Body weight of conventional mice infected with *S*. Tm and supplemented with water or 4.5% (m/v) L-arabinose (L-ara)-containing water. **, *P* < 0.01; ***, *P* < 0.001, Student’s *t* test. (**C**) The CI of the WT and Δ*araA* at day 4 post-infection. (**D**) The CI of the WT and Δ*araE* at day 4 post-infection. (**E**) Hematoxylin and eosin (H&E)-stained colons of mice. The colons were fixed and embedded in paraffin and stained with H&E. Images were taken at ×200 magnification (scale bars, 200 µm). (**F**) Neutrophil infiltration in ceca. The paraffin section was stained with hematoxylin and anti-MPO antibodies by immunohistochemistry. Images were taken at ×200 magnification (scale bars, 200 µm). (**G**) Immunofluorescence staining of *S*. Tm cecal colonization *in vivo*. The ceca were fixed and embedded in paraffin and stained for specific *Salmonella* lipopolysaccharide (LPS) and nuclei. Images were taken at ×200 magnification (scale bars, 200 µm).

To understand the effect of dietary L-arabinose on bacterial colitis, we fed mice with 4.5% (m/v) L-arabinose in drinking water following *S*. Tm administration by oral gavage in conventional mice. This dose of L-arabinose is clinically relevant because human clinical trials show that a 4% L-arabinose addition in sucrose beverages reduces postprandial glucose, and insulin and augments the postprandial increase in the GLP-1 response (13). To our surprise, the survival rate of L-arabinose-treated mice was significantly decreased compared with that of control mice (Fig. 1A). Notably, mice body weight loss was also higher in the L-arabinose-treated group on day 1, day 2, and day 3 (Fig. 1B). Furthermore, the bacterial burden in the liver and spleen showed that the wild-type strain (WT) outcompeted the Δ*araA* strain during L-arabinose administration, which demonstrates that *S*. Tm can exploit L-arabinose as a nutrient (Fig. 1C). The AraE permease is essential for transport of L-arabinose, but a high extracellular L-arabinose concentration (>1%) permits transport by AraE-independent routes (24). The competitive index (CI) value of WT/Δ*araE* was nearly 1, indicating that the L-arabinose concentration in the intestinal lumen was higher than 1% (Fig. 1D).

In addition, histopathological analysis showed increased inflammatory changes and architectural distortion in the colon of L-arabinose-treated mice, including crypt loss, edema, and goblet cell loss (Fig. 1E). Increased polymorphonuclear neutrophil (PMN) infiltration (Fig. 1F) and cecal bacteria (Fig. 1G) supported the more severe colitis phenotype following L-arabinose treatment. Together, these data demonstrated that colitis pathogenesis was enhanced by L-arabinose consumption in *Salmonella*-infected conventional mice.

### Consumption of L-arabinose exhibits increased inflammatory changes in a mouse typhoid model

**Fig 2:**
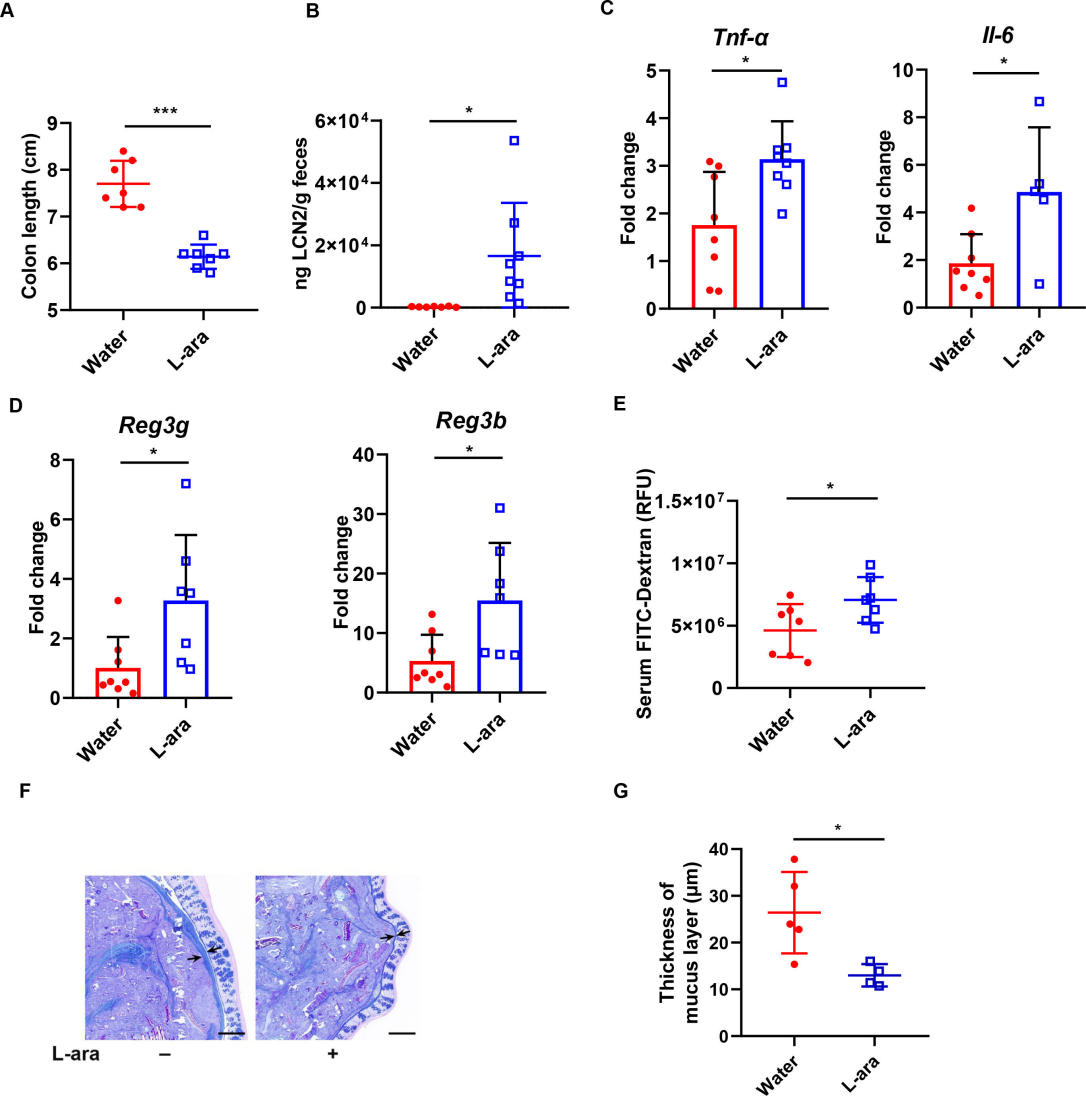
L-arabinose worsens intestinal inflammation in a typhoid mouse model. (**A**) Colon lengths of mice. Mice were sacrificed on day 4, and colon lengths were measured. (**B**) Gut inflammation as measured by Lipocalin-2 enzyme-linked immunosorbent assay (ELISA). Feces were harvested on day 4 and homogenized in phosphate-buffered saline (PBS) for detecting Lipocalin-2. The transcriptional levels of (**C**) *Tnf-α*, *Il-6*, (**D**) *Reg3g*, and *Reg3b* were measured by quantitative PCR (qPCR). Data represent means ± SD. *, *P* < 0.05, Student’s *t* test. (**E**) Intestinal permeability was determined by measuring [fluorescein isothiocyanate (FITC)-Dextran in serum]. *, *P* < 0.05, Student’s *t* test. (**F**) Alcian blue/periodic acid-Schiff (AB-PAS) stained images of colonic sections showing the mucus layers (arrows). Images were taken at ×200 magnification (scale bars, 200 µm). (**G**) Blinded colonic mucus layer measurements from AB-PAS stained sections. Data are presented as means ± SD. *, *P* < 0.05, Student’s *t* test.

Aggravated colitis development in L-arabinose-fed mice led us to examine whether L-arabinose affected gut architecture or physiology. To this end, we evaluated colon lengths and levels of inflammatory markers in conventional mice supplemented with 4.5% (m/v) L-arabinose in drinking water. In agreement, the colon lengths of L-arabinose-treated mice were shorter than those of untreated controls (Fig. 2A). Elevated concentrations of lipocalin-2 (LCN2), a marker of gut inflammation, verified that L-arabinose elicited a serious immune response (Fig. 2B). We also examined the expression of host key genes involved in inflammation, epithelial repair, and innate defense. The expression of proinflammatory cytokines including *Il-6* and *Tnf-α* was higher in the colons of L-arabinose-treated mice on day 4 post-infection (Fig. 2C). Consistently, the transcriptional levels of mouse antibacterial genes *Reg3b* and *Reg3g* were increased by L-arabinose treatment (Fig. 2D). Given the above observations, we concluded that L-arabinose exacerbated intestinal inflammation in *Salmonella*-induced colitis.

Increased gut inflammation is associated with elevated intestinal permeability and impaired tight-junction integrity (25, 26). Therefore, we began our investigation of the drivers of exacerbated enteric inflammation by hypothesizing that L-arabinose leads to intestinal barrier dysfunction. As expected, the FITC-dextran permeability assay showed that gut permeability increased in the L-arabinose-treated mice during *Salmonella* infection (Fig. 2E). The thickness of the colonic mucus layer was measured using Alcian blue/periodic acid-Schiff (AB-PAS) staining. The results revealed that mucus thickness was thinner in L-arabinose-treated mice (Fig. 2F and G).

**Fig 3:**
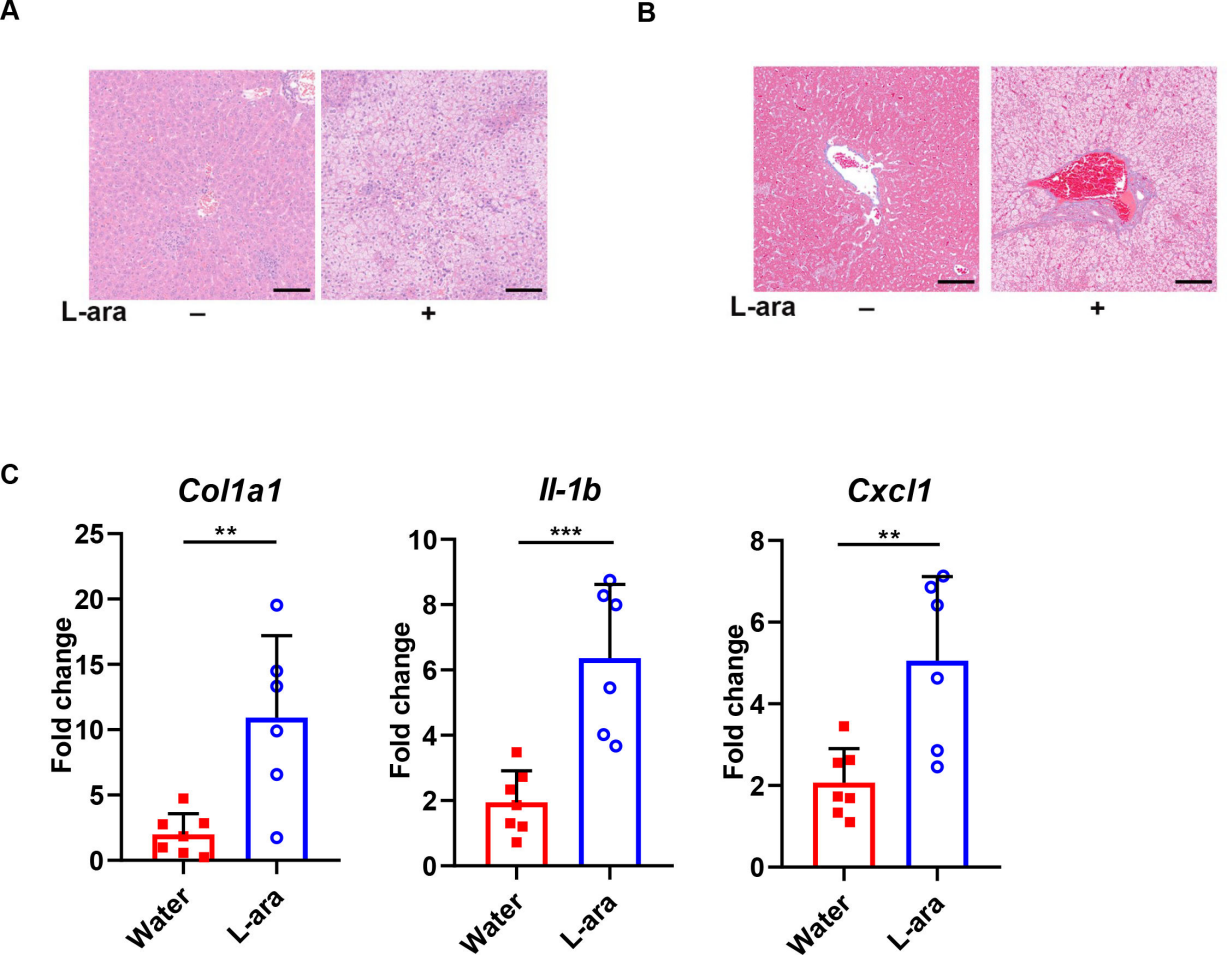
L-arabinose triggers more severe liver inflammation upon *S*. Tm infection. (**A**) H&E, (**B**) Masson staining of liver tissues. The liver tissues were fixed and embedded in paraffin and stained with H&E or Masson’s trichrome. Images were taken at ×400 magnification (scale bars, 100 µm). (**C**) The transcriptional levels of *Col1a1*, *Il-1b*, and *Cxcl1* were measured by qPCR. **, *P* < 0.01; ***, *P* < 0.001, Student’s *t* test.

Alterations in permeability allow gut-derived toxins to cross the intestinal barrier via the gut-liver axis and activate Kupffer cells in the liver, causing hepatic injury and systemic inflammation. Histological analysis using hematoxylin and eosin (H&E) staining showed significant hepatocyte necrosis and a disordered lobule structure in the liver tissues of L-arabinose-treated mice (Fig. 3A). Masson’s trichrome staining also exhibited marked fatty changes with hepatic fibrosis in the L-arabinose-treated group (Fig. 3B). The transcriptional level of *Col1a1* (marker of hepatic fibrosis) was significantly higher in the L-arabinose-treated group (Fig. 3C). L-arabinose-treated mice had more liver inflammation with higher expression levels of mRNAs encoding inflammatory cytokines and chemokines (*Il-1b* and *Cxcl1*) than control mice (Fig. 3C). Collectively, these results further validated that L-arabinose promoted *Salmonella* inflammation and systemic spread in typhoid mice, especially causing fibrosis and inflammation in the liver.

### L-arabinose reconstitutes the composition of *Enterobacteriaceae* in *Salmonella*-infected mouse gut

The above results led to the assumption that *S*. Tm outcompetes commensals to establish host colonization by better utilization of L-arabinose, further triggering exacerbated colitis and systemic infection in typhoid mice. Inconsistently and surprisingly, L-arabinose-treated mice shed significantly less *S*. Tm throughout infection (days 1, 2, and 3 post-infection) (Fig. S1A). In addition, mice supplemented with or without L-arabinose had comparable levels of *S*. Tm colonization in systemic tissues, such as the spleen and liver (Fig. S1B). The above observation suggests that more severe infection in L-arabinose-treated mice was not caused by excessive *Salmonella* multiplication.

**Fig 4:**
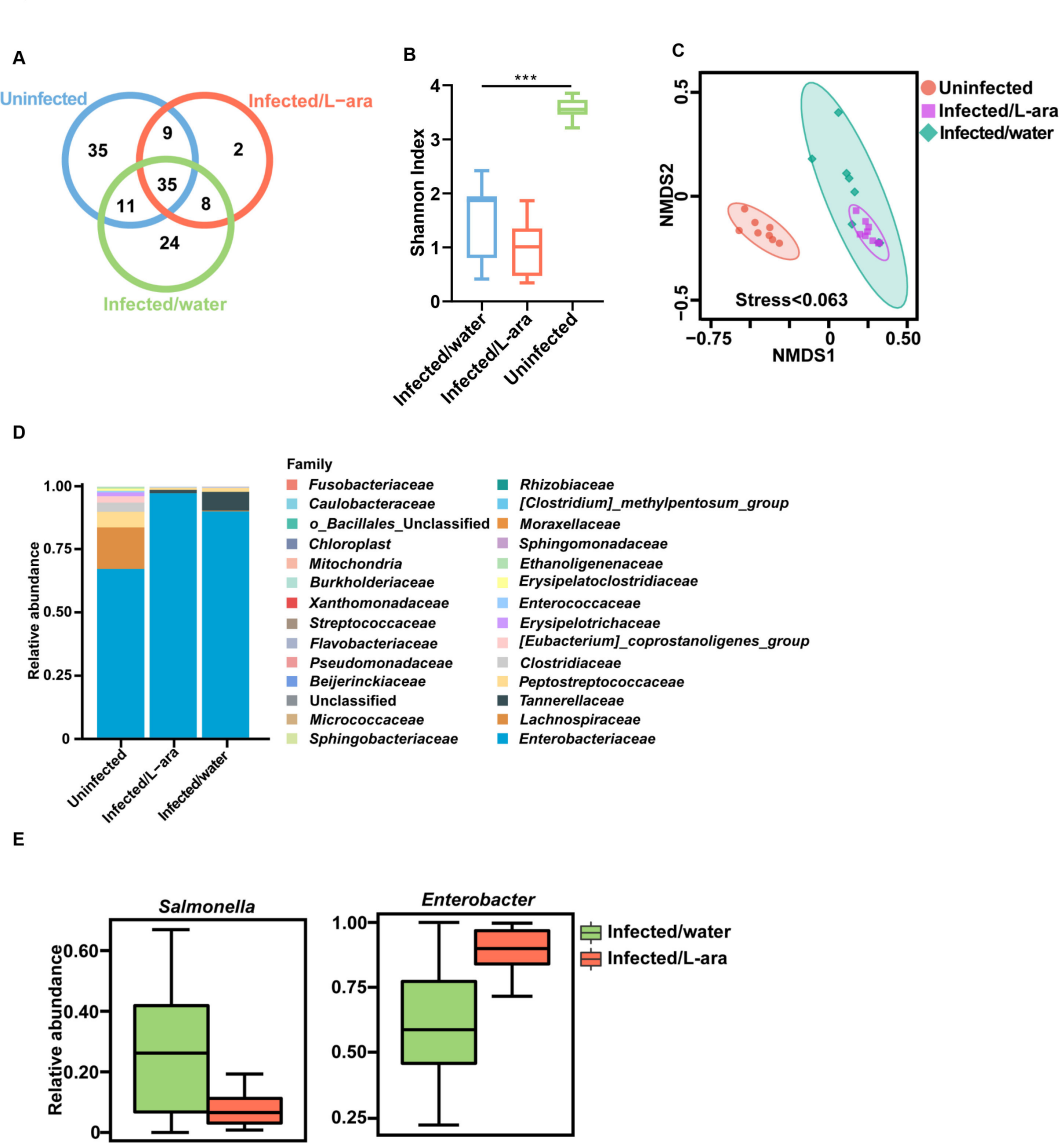
The relative abundance of *Enterobacteriaceae* is enriched in L-arabinose-treated mice. (**A**) The Venn diagrams show OTUs detected in uninfected, L-arabinose-treated (Infected/L-ara), and mock-treated (Infected/water) mice. After *S*. Tm infection, mice were fed with 4.5% (m/v) L-arabinose-containing water or regular water for 4 days. (**B**) The α-diversity (Shannon index) of 16S rDNA sequencing among three groups. Statistical significance was calculated using Kruskal-Wallis. (**C**) The β-diversity (non-metric multidimensional scaling, NMDS) of 16S rDNA sequencing among three groups. (**D**) Relative OTUs abundance of taxonomic distributions at the family level. (**E**) Relative abundance of *Enterobacter* and *Salmonella* in the gut microbiota. Metastats were used to determine differentially abundant bacterial taxa between groups.

Resident microbiota, such as commensal *E. coli* also consumes L-arabinose as a carbon source (5). Cometabolism of L-arabinose by *Salmonella* and commensals is likely to alter their ecological niche and abundance in the intestinal tract, which requires further exploration by 16S ribosomal DNA (rDNA) sequencing. Prior studies implicated that diet shaped the gut microbiota (27, 28), so we hypothesized that L-arabinose altered the gut microbiota composition. Therefore, we profiled the effect of L-arabinose on microbiome composition using 16S rDNA sequencing. The Venn diagram showed that 90 operational taxonomic units (OTUs) were detected in the guts of uninfected mice. Moreover, when mice were infected with *S*. Tm, only 54 OTUs were identified in L-arabinose-treated mice, compared to 78 OTUs in the mock-treated group (Fig. 4A). Specifically, L-arabinose-treated mice presented the lowest α-diversity index in microbiota composition (Fig. 4B). The β-diversity was significantly different following L-arabinose treatment, indicating a shift in the overall gut microbiota composition (stress < 0.063) (Fig. 4C). At phylum level, we observed a significant increase in the relative abundance of Proteobacteria in the L-arabinose-treated group compared with that in the mock-treated group (Fig. S2A; Table S1). Of this phylum, the family of *Enterobacteriaceae* was enriched in the L-arabinose-treated group (Fig. 4D; Table S1). However, L-arabinose-treated mice had a reduction in *Salmonella* (Fig. 4E; Table S2), which was consistent with the fecal shedding results (Fig. S1A).

We also performed a linear discriminant analysis effect size (LEfSe) to identify specific taxa with varied abundance that would potentially be used as biomarkers. In total, we found 43 differentially abundant taxa among the three groups, all of which had a log-linear discriminant analysis score >3. In addition, both the LEfSe result and Metastats analysis validated that *Enterobacter* levels were significantly higher in the L-arabinose-treated group (Fig. 4E; Fig. S2B). Together, these data suggest that the family of *Enterobacteriaceae* bloomed due to L-arabinose supplementation in *Salmonella*-infected mice, limiting the abundance of *Salmonella* and reducing the diversity of the gut microbiota. Such an expansion of specific bacterial taxa and rapid decrease in microbial diversity deteriorated to a state of dysbiosis, further triggering serious inflammatory responses.

### L-arabinose-induced colitis pathogenesis is dependent on microbiota

**Fig 5:**
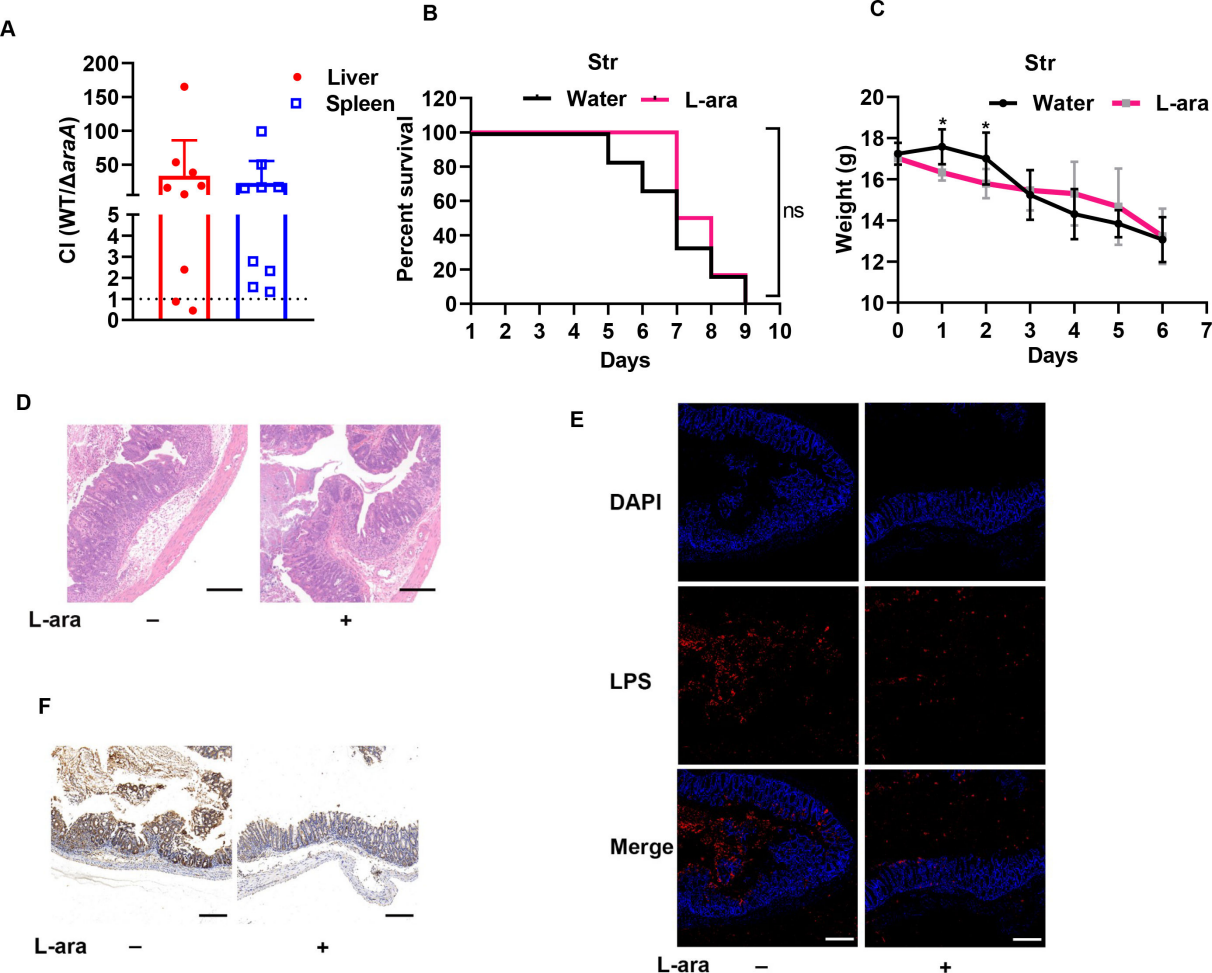
L-arabinose does not influence disease progression in a streptomycin-pretreated mouse model. (**A**) The CI of WT and Δ*araA* at day 4 post-infection. The dotted line indicates equal fitness between WT and mutant. (**B**) Survival curve of streptomycin-pretreated mice infected with *S*. Tm and supplemented with 4.5% (m/v) L-arabinose (L-ara)-containing water or regular water. Ns, *P* > 0.05, log-rank test. (**C**) Body weight of streptomycin-pretreated mice infected with *S*. Tm and supplemented with 4.5% (m/v) L-arabinose (L-ara)-containing water or regular water. *, *P* < 0.05, Student’s *t* test. (**D**) H&E-stained colons of streptomycin-pretreated mice. (**E**) Immunofluorescence staining of *S*. Tm cecal colonization in streptomycin-pretreated mice. (**F**) Neutrophil infiltration in ceca of streptomycin-pretreated mice. The ceca were fixed and embedded in paraffin. Images were taken at ×200 magnification (scale bars, 200 µm).

To determine whether L-arabinose-modulated microbiota was responsible for enhanced colitis pathogenesis, we treated mice with streptomycin to effectively deplete microbial load in the gut. Similarly, the CI values of WT/Δ*araA* by competitive assay were nearly 20 in the liver and spleen in streptomycin-pretreated mice (Fig. 5A), indicating that *Salmonella* was still able to utilize L-arabinose as a carbon source to replicate and cause systemic infections when gut microbiota was absent.

Next, when we infected BALB/c mice pretreated with streptomycin by *S*. Tm and provided L-arabinose-containing water for the duration of the experiment, no significant differences in survival rate (Fig. 5B) or body weight loss were observed (Fig. 5C). In addition, L-arabinose treatment did not affect the severity of colitis pathogenesis in streptomycin-pretreated mice. Mice from both groups exhibited multiple features of colitis with loss of epithelial crypts, inflammation, and edema (Fig. 5D). Moreover, immunofluorescence showed that L-arabinose was unable to inhibit *S*. Tm to colonize cecal tissue at 96 h, although more bacteria could be observed in cecal contents when providing streptomycin-pretreated mice with water (Fig. 5E). Similarly, we also observed comparable levels of PMN infiltration of the submucosa and epithelial layer between the L-arabinose-treated and untreated group, while more PMN infiltration could be observed in the cecal contents of the untreated group at 96 h (Fig. 5F). These results further confirmed that increased colitis pathogenesis in conventionally raised mice was due to L-arabinose-induced alteration of the microbial community in the gut.

To further understand the effect of L-arabinose on *Salmonella* infection, we performed a lethal dose 50 (LD50) assay in the presence and absence of L-arabinose treatment. LD50 of *S*. Tm for conventional mice in the absence of L-arabinose drinking water was found to be 2.37 × 10^5^ colony-forming unit (CFU) as compared to the LD50 of *S*. Tm in the presence of L-arabinose drinking water (6.70 × 10^4^ CFU) (Tables 1 and 2). This resulted in a 3.5-fold decrease in the LD50 indicating L-arabinose increased susceptibility to *Salmonella* infection in conventional mice. Compared to conventional mice, we observed the LD50 of bacteria in streptomycin-pretreated mice with or without L-arabinose treatment was 3.51 × 10^4^ and 1.77 × 10^4^ CFU, respectively (Tables 3 and 4). These data strongly support that pretreatment of conventional mice with antibiotics renders them susceptible to *Salmonella* infection, and this effect is much more remarkable than the treatment of L-arabinose drinking water.

**TABLE 1:** LD50 of *S*. Tm in conventional mice

Challenge dose (CFU)	No. of deaths/total no. of mice	LD50 (CFU)
1 × 10^6^	4/6	2.37 × 10^5^
1 × 10^5^	2/6	
1 × 10^4^	1/6	
1 × 10^3^	0/6	

**TABLE 2:** LD50 of *S*. Tm in conventional mice provided with L-arabinose drinking water

Challenge dose (CFU)	No. of deaths/total no. of mice	LD50 (CFU)
1 × 10^6^	6/6	6.70 × 10^4^
1 × 10^5^	3/6	
1 × 10^4^	1/6	
1 × 10^3^	0/6	

**TABLE 3:** LD50 of *S*. Tm in streptomycin-pretreated mice

Challenge dose (CFU)	No. of deaths/total no. of mice	LD50 (CFU)
1 × 10^6^	6/6	1.77 × 10^4^
1 × 10^5^	4/6	
1 × 10^4^	2/6	
1 × 10^3^	2/6	

**TABLE 4:** LD50 of *S*. Tm in streptomycin-pretreated mice provided with L-arabinose drinking water

Challenge dose (CFU)	No. of deaths/total no. of mice	LD50 (CFU)
1 × 10^6^	6/6	3.51 × 10^4^
1 × 10^5^	3/6	
1 × 10^4^	2/6	
1 × 10^3^	1/6	

### L-arabinose has no short-term effect on hyperglycemia by *Salmonella* infection

**Fig 6:**
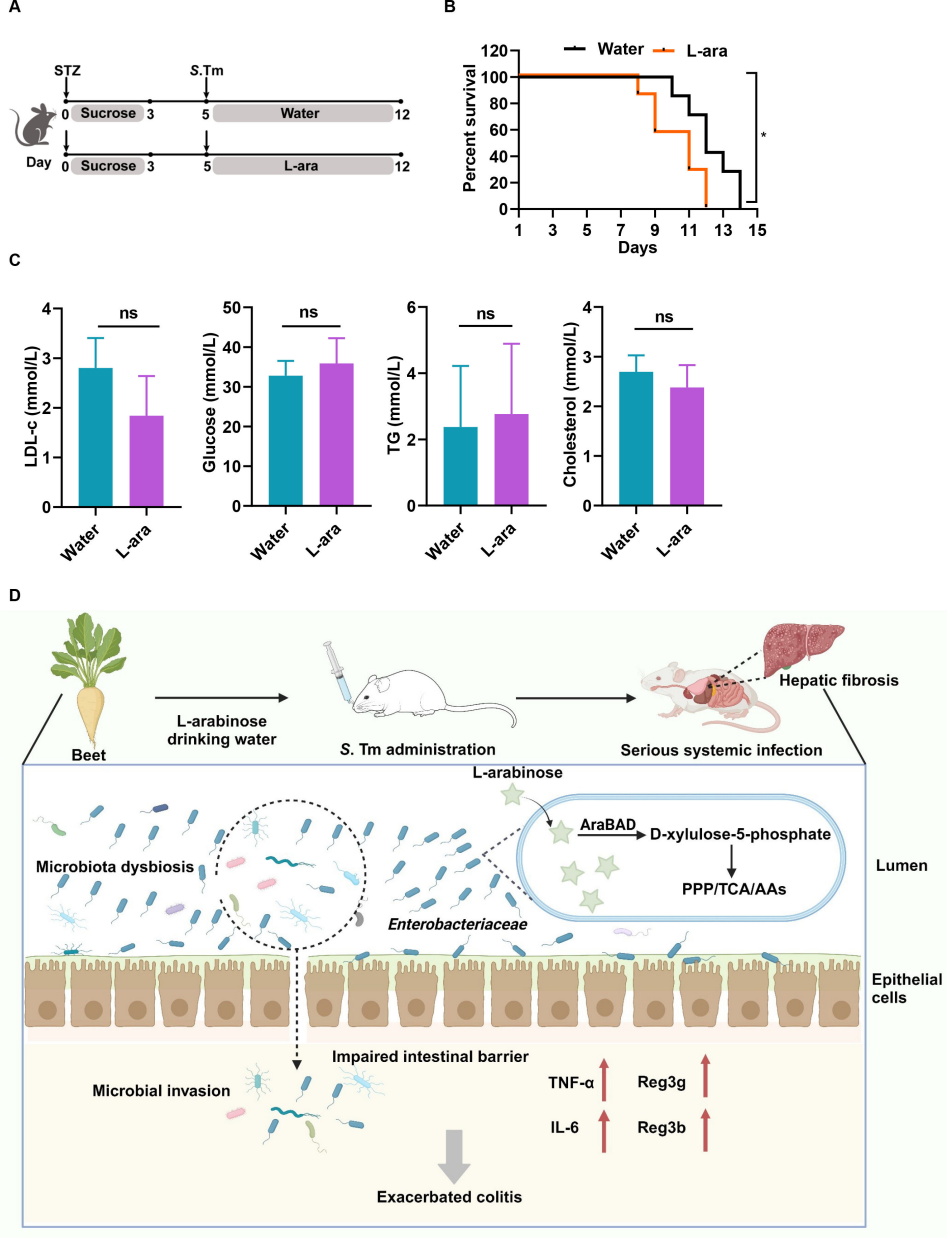
Short term intake of L-arabinose is unable to alleviate symptoms of hyperglycemia and exacerbates negative impact on survival in *Salmonella*-infected mice. (**A**) Experimental design of *Salmonella* challenge study in STZ-induced hyperglycemia model. (**B**) Survival curve of STZ-induced mice infected with *S*. Tm and supplemented with water or 4.5% (m/v) L-arabinose (L-ara)-containing water. *, *P* < 0.05, log-rank test. (**C**) Serum level of low-density lipoprotein cholesterol (LDL-c), glucose, total serum cholesterol (TG), and cholesterol at day 12. Data are presented as the means ± SD. Ns, *P* > 0.05, Student’s *t* test. (**D**) Working model displays the role of L-arabinose supplement in *Salmonella* infection. The proposed scheme shows that L-arabinose induces the blooming of *Enterobacteriaceae* and subsequently triggers microbiota dysbiosis upon *Salmonella* infection, disrupting the intestinal barrier in response to epithelial cell damage. Hyperpermeability of the gut leads to increased bacterial translocation and inflammatory responses and promotes systemic infection.

Our above results revealed that L-arabinose exacerbated colitis and hepatic fibrosis upon *Salmonella* infection in a typhoid mouse model. However, prior studies reported that L-arabinose improved insulin sensitivity and glucose uptake in both animals and humans (29, 30), suggesting that L-arabinose is a potential candidate for combating sucrose-related human pathologies. In addition, the relative abundance of *Enterobacteriaceae* is significantly associated with the severity of type 2 diabetes mellitus (31, 32). As a result, we were curious whether L-arabinose increased the susceptibility to *Salmonella* infection in hyperglycemic mice. To address this concern, we profiled the impact of L-arabinose on the *Salmonella*-infected hyperglycemic mouse model induced by streptozotocin (STZ). Mice were checked for hyperglycemia on day 5 post-STZ injection and then randomized for *Salmonella* infection (Fig. 6A). Mice started to die 10 days post-infection, and all died within 14 days when water was provided *ad libitum*. In contrast, mice supplied with L-arabinose-containing water started to die on day 8 post-infection, and all died within 12 days (Fig. 6B). The results demonstrated that L-arabinose also aggravated *Salmonella* infection outcome in the STZ-induced hyperglycemic mouse model.

Given that chronic exposure to L-arabinose has been evidenced to exert anti-diabetic properties *in vivo*, we explored whether a high dose of L-arabinose supplementation ameliorates hyperglycemia and insulin resistance in *Salmonella*-infected mice. No significant differences were observed in total serum cholesterol (TG), triglyceride, low-density lipoprotein cholesterol (LDL-c), and blood glucose levels after 1 week of L-arabinose treatment (Fig. 6C).

## DISCUSSION

Pathogens utilize a variety of strategies to colonize the gut and cause infection, such as scavenging nutrients, sensing chemical signals, competing with commensals, and regulating the expression of virulence genes (33). Several studies have elucidated the role of a certain carbon source in enteric infections (3, 34). In this study, we used *in vivo* experiments to decipher the role of L-arabinose supplementation in *Salmonella* infection. Here, we report that excessive L-arabinose supplementation accelerates the disease progression of *Salmonella* infection. In conventional mice, gut microbiota plays a central role in maintaining colonization resistance, because streptomycin treatment decreased LD50 to nearly 10-fold as shown in Tables 1 and 3. As an enteropathogen, *S*. Tm overcomes colonization resistance by inducing host inflammatory immune responses, which changes the nutritional environment of the gut lumen for niche creation and nutrient utilization. Exogenous supplementation of dietary L-arabinose facilitates commensals for catabolism, which promotes the rapid emergence of *Enterobacteriaceae*. The bloom of potentially harmful Proteobacteria, especially *Enterobacteriaceae*, triggers microbiota dysbiosis and a compromised intestinal barrier. *Enterobacteriaceae* may possess a more immunostimulatory version of lipopolysaccharide (LPS) (35), which interacts with receptors on immune cells to trigger inflammation. Hyperpermeability of the gut leads to intestinal bacterial translocation, further exacerbating local or systemic inflammatory and immune responses (Fig. 6D).

Although it has been predicted that a high-sugar diet is a risk factor for gastrointestinal diseases, the precise mechanism is poorly understood. This study suggests a mechanism that might explain the association of high-L-arabinose intake with exacerbated *Salmonella* infection. By contrast, this finding is inconsistent with that of a previous study showing that L-arabinose supplementation alleviated colitis in inflammatory bowel disease (IBD) mice (36). Firstly, we believe this discrepancy in the effect of L-arabinose on colitis may be explained by dosage. A recent study demonstrated that the effect of a chemical compound was exerted in a dose-dependent manner, as a high intake of soluble fiber could induce gut dysbiosis and contribute to colorectal tumorigenesis (37). The dose of L-arabinose used in our study is 4.5% (m/v), which is equivalent to 0.18–0.31 g daily per mouse. In comparison, L-arabinose was given daily (400 mg/kg body weight) by Li et al., which is about 0.8 mg daily per mouse. Secondly, Li et al. used dextran sodium sulfate (DSS) to induce colitis, which is a chemical colitogen to induce epithelial damage. Inconsistently, we applied *Salmonella* to trigger intestinal inflammation. The competition for space and nutrition favored the overgrowth of *Enterobacteriaceae*, further leading to gut dysbiosis. However, Li et al. reported that L-arabinose maintained the level of anti-inflammatory *Actinobacteria* in DSS-induced colitis. Altogether, these findings therefore suggest that both the dosage and mouse model may influence the effect of L-arabinose on colitis.

### *Enterobacteriaceae* expansion is a common feature of dysbiosis

Inhibition of pathogens colonization by the resident microbiota, a process that is also called “colonization resistance,” plays an essential role in maintaining animal and human health (38). The composition of the microbiota is profoundly affected by diet, changing the host’s susceptibility to infection (39). Differential dietary sugar availability influences the relationship between microbiota and enteric pathogens. Streptomycin-pretreated mice are mostly used to study colitis caused by *Salmonella* infections (40). However, disruption of the commensal community in this model is unable to investigate the relationship between pathogens and microbiota during enteric infections, and such models are not fully reminiscent of typhoidal salmonellosis in humans (41–43). Interestingly, we found that L-arabinose limited the bloom of *S*. Tm as early as 24 h (Fig. S1A). This effect was ascribed to the specific composition of the microbiota induced by L-arabinose treatment. Moreover, the microbial dissimilation of L-arabinose promotes intermediate metabolite production, such as acetate, lactate, propionate, and succinate (15, 17). Competition for L- arabinose as well as short-chain fatty acid (SCFAs) shapes the environment of the colon in many ways, ultimately impacting disease outcomes. Supplementation with L-arabinose increased the relative abundance of *Enterobacteriaceae*, this phenomenon was caused by the special microbiota community of healthy BALB/c mice, as well as nutritional competition after *S*. Tm infection. While Proteobacteria and Firmicutes constitute the microbiota of healthy mice, Bacteroides were not detected in healthy mice by 16S rDNA sequencing. The unique bacterial community structure in BALB/c mice provides an explanation for this phenomenon because the main phylum is determined to be Firmicutes, as reported by others (44).

Proteobacteria bloom is a characteristic trait of abnormal microbiota in the course of antibiotic therapy and dietary changes (45). In addition, microbiota dysbiosis is commonly observed in patients with IBD, Crohn’s disease, and ulcerative colitis, which are characterized by an increased relative abundance of facultative anaerobic bacteria (e.g., *Enterobacteriaceae* and *Bacilli*) (46, 47). More importantly, dysbiosis disrupts the mucosal barrier, resulting in the perpetuation of inflammation and carcinogenesis (48, 49). Conversely, inflammatory disease conditions favor the overgrowth of pathogens and specific commensal species, in particular the family members of the *Enterobacteriaceae* (45, 50), which leads to a vicious cycle of enteric infections.

### Disturbed microbiota are associated with gut barrier dysfunction and inflammation

Recent studies suggest that a sucrose-rich diet and a fructose-rich diet lead to a reduction of microbial diversity, which, in turn, increases intestinal permeability and activates host inflammation (51, 52). Although it has been predicted that a high-sugar diet is a risk factor for colitis, the underlying mechanism is poorly understood. Here, we demonstrate that diets including high L-arabinose reduced the thickness of the mucus layer (Fig. 2E through G), allowing intestinal bacteria to come near the epithelial layer. Furthermore, a potential finding of our study is that L-arabinose may drive inflammation in *Salmonella*-infected mice (Fig. 2C and 3C). The gut microbiota is responsible for regulating the integrity and function of the gut barrier in a homeostatic balance. Several studies have shown that gut dysbiosis leads to epithelial barrier dysfunction (53). Dysfunction of gut barrier integrity may enable bacteria and their toxic metabolites, such as LPS to cross the intestinal barrier and enter systemic circulation, which interacts with Toll-like receptors to induce systemic inflammation (54). Intestinal hyperpermeability leads to “leaky gut syndrome” (55), which supports our findings that L-arabinose-induced microbiota dysbiosis may trigger gut barrier dysfunction and inflammation.

While our 16S rDNA sequencing robustly characterizes the microbiome, there are limitations in the study design and methodology that complicate the interpretation of the results. Firstly, we fail to identify the drivers of increased inflammation in conventional mice by L-arabinose treatment. Secondly, the mechanism of *Enterobacteriaceae* enrichment during L-arabinose intake and its role in colitis pathogenesis still need further investigation. Ultimately, further work will be required to correlate L-arabinose and inflammatory changes with individual taxa of the microbiome. Specifically, subsequent metabolomics analyses will be critical for identifying the metabolites contributing to the phenotype we observed by L-arabinose treatment in conventional mice.

### L-arabinose poses a potential risk for diabetics with enteric infections

Sugar overconsumption is linked to a rise in the incidence of diseases such as diabetes, cardiovascular diseases, and gastrointestinal diseases. For example, high glucose intake exacerbates the pathogenesis of autoimmunity and inflammation (56). It has been reported that elevated consumption of fructose worsens DSS-induced or infectious colitis (57). In addition to these caloric simple sugars, a randomized-controlled clinical trial showed that non-nutritive sweeteners, saccharin, and sucralose distinctly alter the stool microbiome and impair glycemic responses (58).

L-arabinose was approved as generally recognized as safe by the U.S. Food and Drug Administration in 2018. Supplementation with L-arabinose has been shown to significantly improve glucose intolerance and gut microbiota incoordination in diabetes (30, 59). However, we found that non-caloric L-arabinose exacerbated infectious colitis by altering microbiota composition. Some side effects, such as stomachache and diarrhea are associated with the addition of L-arabinose (13), which may correlate with bacterial infections for diabetics. Since hyperglycemia affects both microbiome structure and metabolism (60), we assume that the synergistic effect exerted by both L-arabinose and hyperglycemia further exacerbates colitis.

A limitation of this study is that it was conducted in an animal model. Therefore, the effects of L-arabinose on *Salmonella* infection need to be validated in clinical settings. Furthermore, we do not have the necessary tool-set to study the mechanism of effects between L-arabinose and *Salmonella* infection on hyperglycemia mice. The exact role of L-arabinose during *Salmonella* infection in hyperglycemia mice is unknown but should be investigated in future studies.

## MATERIALS AND METHODS

### Bacterial strains, plasmids, media, and culture conditions

Streptomycin resistance *S*. Tm 14028S-str was used for mouse infection. Strains were grown in LB medium supplemented with antibiotics as required. Solid media were prepared by the addition of 1.5% agar. The final concentration of streptomycin in media was 50 µg/mL.

### RNA isolation, sequencing, and quantitative real-time PCR assay

Mouse tissue RNA was extracted using TRIzol according to the manufacturer’s instructions. RNA samples were quantified and reverse-transcribed with random hexamers. Quantitative real-time PCR was carried out in 20 µL of reaction mixture using ChamQ SYBR Color qPCR Master Mix (Vazyme). Fold changes in gene expression were calculated based on the 2^−ΔΔCt^ method.

### Mouse experiments

Female 6–8-week-old BALB/c mice were used in this study. Mice were housed under specific pathogen-free conditions and maintained on a 12-h light/dark cycle. Food and water were provided *ad libitum*. Twenty milligrams of streptomycin were administered by oral gavage per day before infection when necessary. The infected mice were monitored daily for signs of clinical illness.

Mice were infected by oral gavage with 100 µL of phosphate-buffered saline (PBS) containing approximately 1.5 × 10^7^ CFU of *S*. Tm. Sterile drinking water containing 4.5% (m/v) L-arabinose was provided *ad libitum* during the whole infectious process. The survival of the mice was observed daily after infection.

In a competitive infection assay, two competitive *S*. Tm strains at a ratio of 1:1 were given via oral gavage at a combined final concentration of 1.5 × 10^7^ CFU/mouse. For CFU determinations, the spleen and liver were collected and plated in ten-fold serial dilutions at day 4 post-infection. To determine the bacterial numbers in the feces, fecal pellets were collected individually from mice, weighed, homogenized in cold PBS, and plated at serial dilutions onto LB agar. Ceca and colons were harvested, fixed, and processed for H&E staining and immunohistochemistry. Mice were sacrificed on day 4 post-infection, and colon content and tissue were collected for 16S rDNA sequencing and RT-qPCR analysis.

### Streptozotocin-induced diabetic mouse model

A single, high dose of STZ was utilized to induce a type 1 diabetic model in C57BL/6 male mice (61). To induce hyperglycemia, STZ was diluted freshly in 50 mM sodium citrate buffer (pH 4.5) and injected intraperitoneally at 200 mg/kg. Drinking water containing 10% sucrose was provided until experimental day 3 to avoid post-procedural hypoglycemia. Five days post-injection, fasting blood glucose was tested from the tail vein by using One Touch Basic Blood Glucose Monitoring System (Roche). Mice (fasting blood glucose ≥ 8.3 mmol/L) were selected for subsequent bacterial infection. For *Salmonella* infection, mice were infected by oral gavage with 100 CFU of strain 14028S-str, and serum was collected on day 12.

### Lipocalin-2 quantification

Fresh stool samples from the mice were collected in tubes and frozen in lipid nitrogen immediately. Feces were homogenized in 1 mL of sterile PBS and supernatants were collected to detect lipocalin-2 by Mouse NGAL enzyme-linked immunosorbent assay (ELISA) Kit (Proteintech, Chicago, IL, USA).

### Colonic epithelial barrier permeability measurement by FITC-dextran

Mice were given 150 µL of FITC-Dextran (4 kDa) suspension (80 mg/mL) by gavage on day 4 post-infection. After 3 h, blood was collected and centrifuged at 4°C. The fluorescence signal of serum was quantified at 485/525 nm (excitation/emission).

### Extraction of genomic DNA and 16S rDNA sequencing

The total genomic DNA of fecal samples was extracted using the QIAamp Fast DNA Stool Mini Kit (Qiagen, Hilden, Germany). The V3-V4 hypervariable regions of the 16S rRNA gene were amplified using the forward primer (CCTACGGRRBGCASCAGKVRVGAAT) and the reverse primer (GGACTACNVGGGTWTCTAATCC). Next-generation sequencing was conducted on an Illumina Novaseq platform. Raw sequence data were quality-filtered, followed by VSEARCH clustering (1.9.6) for OTUs clustering. The OUTs were classified taxonomically using the Ribosomal Database Program classifier and calculated the relative abundance of microbiota at different levels. The Shannon index was obtained using a random sampling method. The non-metric multidimensional scaling (NMDS) was based on the distance between the matrix Brary-Curtis to display β-diversity. The LEfSe scores measure the consistency of differences in relative abundance between taxa in the groups, with a higher score indicating higher consistency.

### LD50 assay

The LD50 assay was carried out as described in previous studies (62). Briefly, two separate groups of conventional mice (each containing 24 mice) were infected with *S*. Tm with or without L-arabinose drinking water. Similarly, two separate groups of streptomycin-pretreated mice (each containing 24 mice) were infected with *S*. Tm with or without L-arabinose drinking water. Mice in each group were further subdivided into four subgroups each containing six mice. The subgroups were given intragastric administrations of *S*. Tm at doses ranging from 1 × 10^6^ to 1 × 10^3^ CFU/mouse. The LD50s were calculated 14 days post-infection by the method of Reed and Muench (63).

### Statistical analysis

All the data were collected from more than three independent experiments. Data were analyzed using the GraphPad Prism 8.0 software (GraphPad Software). Statistically significant differences were examined using a two-tailed Student’s *t* test or Mann-Whitney test to derive the significance of differences between the two groups. One-way analysis of variance test was used for multiple comparisons. Log-rank test was used for comparing survival distributions between groups. Shannon index was compared with the Kruskal-Wallis test. *P* < 0.05 was considered to be significant.

## Data Availability

The authors confirm that the data supporting the findings of this study are available within the article and its supplementary materials.

## SUPPLEMENTAL MATERIAL

Figure S1 msystems.00522-24-s0001.tif L-arabinose inhibits *Salmonella* expansion.

Figure S2 msystems.00522-24-s0002.tif Comparison of gut microbiomes.

Supplemental Text File msystems.00522-24-s0003.pdf Supplemental legends

Table S1 msystems.00522-24-s0004.xlsx Relative OTUs abundance of taxonomic distributions between three groups.

Table S2 msystems.00522-24-s0005.xlsx Significant different taxonomic composition at genus level analyzed by Metastats.

Table S3 msystems.00522-24-s0006.xlsx PCR primers used in this study.

ASM does not own the copyrights to Supplemental Material that may be linked to, or accessed through, an article. The authors have granted ASM a non-exclusive, world-wide license to publish the Supplemental Material files. Please contact the corresponding author directly for reuse.
