# Supplementary figures and images for: Dietary L-arabinose-induced gut dysbiosis exacerbates Salmonella infection outcome
Source: mSystems. 2024 Jul 9;9(8):e00522-24. doi: 10.1128/msystems.00522-24 (PMC11334454; doi:10.1128/msystems.00522-24)

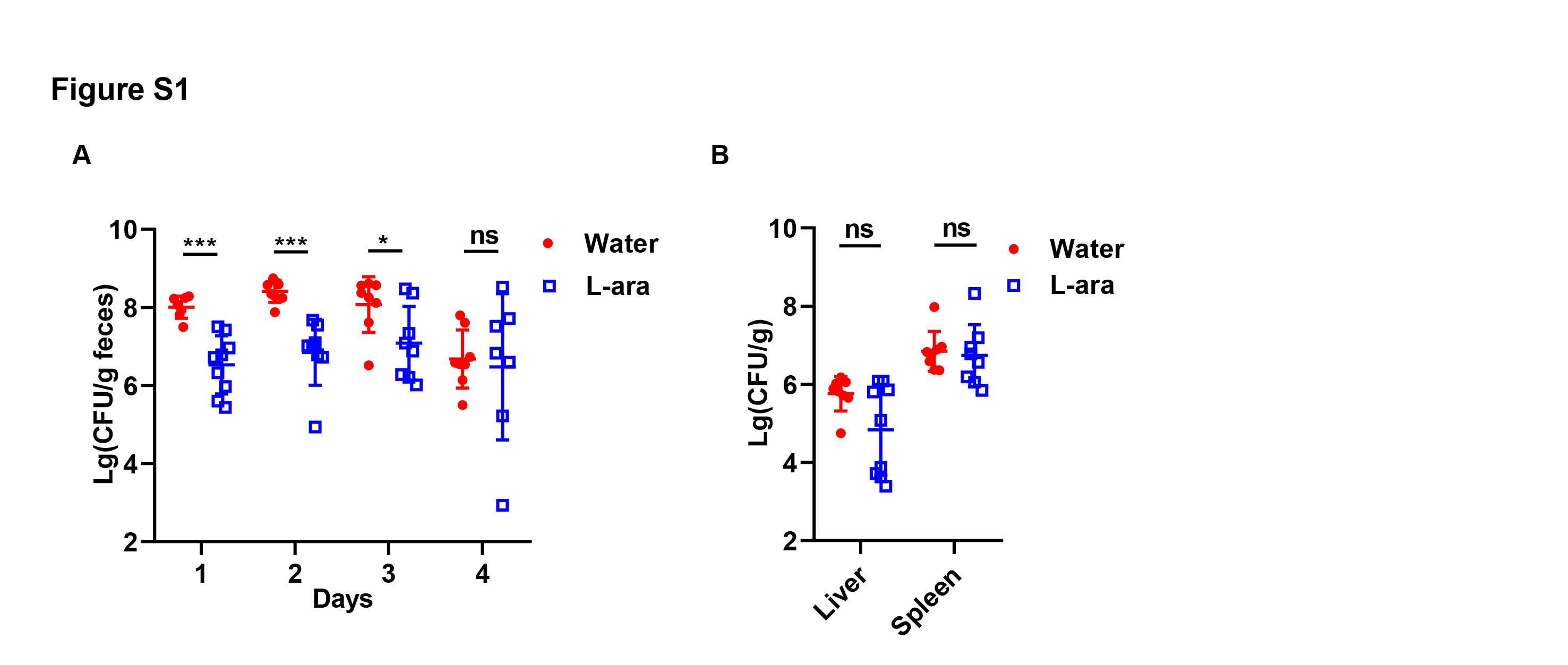

Supplement: Figure S1 — L-arabinose inhibits Salmonella expansion. [file msystems.00522-24-s0001.tif]

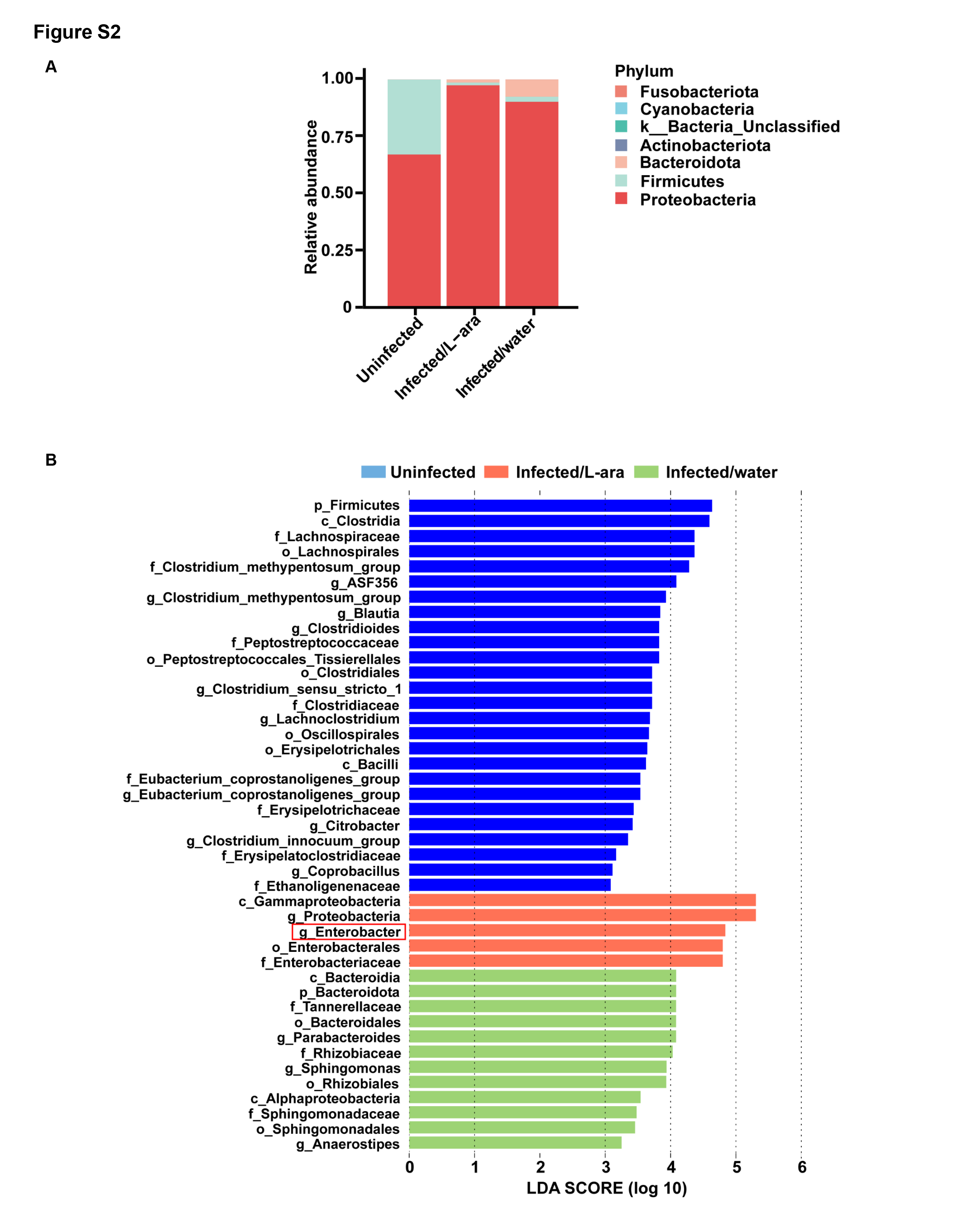

Supplement: Figure S2 — Comparison of gut microbiomes. [file msystems.00522-24-s0002.tif]
