# Supplementary material for: Dietary L-arabinose-induced gut dysbiosis exacerbates Salmonella infection outcome
Source: mSystems. 2024 Jul 9;9(8):e00522-24. doi: 10.1128/msystems.00522-24 (PMC11334454; doi:10.1128/msystems.00522-24)
Supplement: Supplemental Text File — Supplemental legends [file msystems.00522-24-s0003.pdf]

Supplemental figure legend

Figure S1

L-arabinose inhibits expansion of *S*. Tm in the gut lumen.

(**A**) Fecal shedding of mice. Mice were infected with *S*. Tm and supplemented with water or 4.5% (m/v) L-arabinose (L-ara)-containing water. Feces were quantified over 4 days. *, $P < 0.05$, ***, $P < 0.001$, by two-tailed Mann-Whitney test. (**B**) Tissue burdens at day 4 post- infection. The livers and spleens were harvested and counted. Ns, $P > 0.05$, by two-tailed Mann-Whitney test.

Figure S2

Comparison of gut microbiome reveals biomarkers between L-arabinose-treated and untreated mice.

(**A**) Relative OTUs abundance of taxonomic distributions at phylum level in uninfected, L-arabinose-treated (Infected/L-ara), and untreated (Infected/water) mice. (**B**) LEfSe analysis shows differentially abundant taxa as biomarkers between L-arabinose-treated and untreated mice.

Supplementary tables

Table S1 Relative OTUs abundance of taxonomic distributions between three groups. The file gives the comparison on the mean relative abundance of the community composition in uninfected, L-arabinose-treated (Infected/L-ara), and untreated (Infected/water) mice.

Table S2 Significant different taxonomic composition at genus level analyzed by Metastats. The file summarizes different genus level bacteria abundance between L-arabinose-treated (Infected/L-ara) and untreated (Infected/water) mice. $P<0.05$ denotes statistical significance.

Table S3 PCR primers used in this study.
